# Biosynthesized Silver Nanoparticles Derived from Probiotic *Lactobacillus rhamnosus* (AgNPs-LR) Targeting Biofilm Formation and Quorum Sensing-Mediated Virulence Factors

**DOI:** 10.3390/antibiotics12060986

**Published:** 2023-05-31

**Authors:** Amir Mahgoub Awadelkareem, Arif Jamal Siddiqui, Emira Noumi, Syed Amir Ashraf, Sibte Hadi, Mejdi Snoussi, Riadh Badraoui, Fevzi Bardakci, Mohammad Saquib Ashraf, Corina Danciu, Mitesh Patel, Mohd Adnan

**Affiliations:** 1 Department of Clinical Nutrition, College of Applied Medial Sciences, University of Ha’il, Ha’il P.O. Box 2440, Saudi Arabia; 2 Department of Biology, College of Science, University of Ha’il, Ha’il P.O. Box 2440, Saudi Arabia; 3 Department of Forensic Sciences, Naif Arab University for Security Sciences, Riyadh, Saudi Arabia; 4 Department of Medical Laboratory Science, College of Applied Medical Sciences, Riyadh ELM University, Riyadh, Saudi Arabia; 5 Department of Pharmacognosy, Faculty of Pharmacy, “Victor Babes” University of Medicine and Pharmacy, 2 Eftimie Murgu Square, 300041 Timisoara, Romania; 6 Department of Biotechnology, Parul Institute of Applied Sciences, Centre of Research for Development, Parul University, Vadodara 391760, India

**Keywords:** quorum sensing, biofilms, *Lactobacillus rhamnosus*, *Chromobacterium violaceum*, *Serratia marcescens*, green antimicrobial agent

## Abstract

In recent years, bacterial pathogens have developed resistance to antimicrobial agents that have created a global threat to human health and environment. As a novel approach to combating antimicrobial resistance (AMR), targeting bacteria’s virulent traits that can be explained by quorum sensing (QS) is considered to be one of the most promising approaches. In the present study, biologically synthesized silver nanoparticles derived from *Lactobacillus rhamnosus* (AgNPs-LR) were tested against three Gram-negative bacteria to determine whether they inhibited the formation of biofilms and triggered the virulence factors controlled by QS. In *C. violaceum* and *S. marcescens*, a remarkable inhibition (>70%) of QS-mediated violacein and prodigiosin production was recorded, respectively. A dose-dependent decrease in virulence factors of *P. aeruginosa* (pyocyanin, pyoverdine, LasA protease, LasB elastase and rhamnolipid production) was also observed with AgNPs-LR. The biofilm development was reduced by 72.56%, 61.70%, and 64.66% at highest sub-MIC for *C. violaceum*, *S. marcescens* and *P. aeruginosa*, respectively. Observations on glass surfaces have shown remarkable reductions in biofilm formation, with less aggregation of bacteria and a reduced amount of extra polymeric materials being formed from the bacteria. Moreover, swimming motility and exopolysaccharides (EPS) was also found to reduce in the presence of AgNPs-LR. Therefore, these results clearly demonstrate that AgNPs-LR is highly effective in inhibiting the development of biofilms and the QS-mediated virulent traits of Gram-negative bacteria. In the future, AgNPs-LR may be used as an alternative to conventional antibiotics for the treatment of bacterial infections after careful evaluation in animal models, especially for the development of topical antimicrobial agents.

## 1. Introduction

As a global public health issue, antimicrobial resistance (AMR) is a growing problem that occurs when microorganisms such as bacteria, viruses, fungi, and parasites become resistant to antimicrobial drugs that have previously served as effective treatments for infections [1,2]. When bacteria become resistant to antibiotics, they can spread infections which are difficult to treat, which can result in prolonged illness, disability and even death as a result [3,4]. Multi-drug resistance (MDR) is a form of AMR in which microorganisms become resistant to multiple drugs, making it more difficult to treat infections [5]. This can happen when antibiotics are overused or misused, as well as when there is poor infection prevention and control in healthcare settings [6]. The current problem of AMR and MDR poses a serious problem to global health [7]. According to the World Health Organization (WHO), by 2050 if no action will be taken against drug-resistant infection, the number of deaths will rise to 10 million annually [8]. AMR and MDR also have significant economic impacts. A drug-resistant infection costs more to treat than a non-resistant infection due to the need for more expensive drugs and longer hospital stays [5]. AMR also affects agricultural productivity as it can lead to the loss of livestock and crops [9]. Efforts to address AMR and MDR require a multi-sectoral approach, including reducing unnecessary antibiotic use, improving prevention of infection and measures of prevention, developing new antimicrobial drugs and diagnostic tools, and promoting global cooperation and coordination [10,11,12].

As a strategy to combat the AMR epidemic, anti-infective drugs should be designed, which can target quorum sensing (QS)-regulated virulence factors and biofilm formation [13]. Quorum sensing is a process used by bacteria to communicate with one another through the release and detection of chemical signals called autoinducers. This process permits bacteria to coordinate their behaviour and synchronize their gene expression against the changed environmental conditions [14]. Biofilms are communities of microorganisms embedded in a protective extracellular matrix that adhere to surfaces. QS has a key role in the formation and maintenance of these communities. The eradication of biofilms is extremely difficult and they contribute to the development of antimicrobial resistance by providing a protective environment for bacteria to grow and exchange genetic material, including antibiotic resistance genes [15]. Quorum sensing inhibitors (QSIs) are compounds that can disrupt the communication process between bacteria and prevent the formation of biofilms and reducing the spread of bacterial infections. QSIs have shown promise as a potential strategy to inhibit AMR by reducing resistance to antibiotics in biofilms. Additionally, by preventing the formation of biofilms, QSIs can make bacteria more susceptible to antibiotic treatment, lowering the likelihood of the development of resistance. Several natural and synthetic QSIs have been identified and research is ongoing to develop more effective QSIs and explore their potential clinical applications [16,17]. However, in this regard it is imperative to note that QSIs alone are unlikely to be a promising one for addressing AMR and that a comprehensive approach that includes reducing unnecessary antibiotic use and improving infection prevention and control measures is necessary to combat this global public health challenge [18,19].

Recently, a lot of attention has been paid in finding ways to produce and use the nanomaterials, and the interest is growing every day. In terms of manufacturing nanoparticles, one of the methods to be considered is the bio-approach (green) [20]. Using microorganisms for synthesis of nanoparticles, for example, is referred to as nanoparticles synthesis by biological means. Nanoparticles are produced both by living and dead microorganisms, contribute greatly to nanoparticle production [21]. Material can be controlled at the molecular level using nanotechnology. Silver nanoparticles resulted in significant attention because of their antimicrobial properties, and these metal nanoparticles are being used in different fields such as industrial packaging, agriculture, medicine, cosmetics, and in the military [22]. An array of microorganisms such as *E. coli*, *S. aureus*, *P. aeruginosa*, *B. subtilis*, *V. cholera* and *S. typhus* may be susceptible to AgNPs as antimicrobial agents [22,23,24]. Hence, as an emerging method for discovering antibacterial involves using green synthesized nanoparticles to target bacterial biofilm and QS. Utilization of silver nanoparticles as an alternative antimicrobial agent has been suggested in previous studies and it may prove useful as an alternative [17]. Furthermore, it has become apparent in recent years that nanotechnology has grasped a lot of attention from scientists because of the possibility of its application to medicine, diagnostics, agriculture, bioremediation and many other fields [18]. Nano-scaled materials appear to exhibit better biological effects than their bulk counterparts because their chemical and physical properties are different at this scale, which is mainly why the nano-scaled materials shown improved biological activity [23]. In the future, nanotechnology is expected to have potential applications in different fields of health care, such as for the purposes of new drugs, for drug delivery and diagnostics, and for the creation of improved biomaterials [24].

*Lactobacillus rhamnosus* (*L. rhamnosus*) is a probiotic bacterium that has gained considerable attention in the field of medicine due to its potential health benefits. As a naturally occurring bacterium in the human gastrointestinal tract, *L. rhamnosus* has been extensively studied for its various applications in promoting and supporting human health. With its ability to positively influence the gut microbiota and modulate the immune system, *L. rhamnosus* has shown promising potential in the prevention and management of several medical conditions [25]. This strain is therefore given more importance nowadays for its health benefits, ability to defeat intestinal pathogens, maintain intestinal flora balance, and maintain intestinal barriers [26,27]. The strain also produced antimicrobial metabolites that had an antagonistic effect on harmful bacteria, including *E. coli* [28], *S. enterica* [29] and *S. aureus* [28]. In addition to this, several studies have also shown that *L. rhamnosus* has the ability to reduce the bioavailability of mycotoxins in the gastrointestinal tract as well [30]. Therefore, in the present study, *L. rhamnosus* was used in order to synthesize silver nanoparticles (AgNPs-LR). The synthesized AgNPs-LR were investigated for their broad-spectrum effect on inhibiting the virulence factors controlled by QS in bacterial pathogens namely, *C. violaceum*, *P. aeruginosa* and *S. marcescens* in conjunction with the suppression of biofilm development.

## 2. Materials and Methods

### 2.1. Strains of Bacteria and Growth Conditions

The strain of lactic acid bacteria (LAB), *Lactobacillus rhamnosus* MTCC-1423 (*L. rhamnosus*) and pathogenic Gram-negative bacterial strain *C. violaceum* MTCC-2656 (*C. violaceum*) *P. aeruginosa* MTCC-741 (*P. aeruginosa*) and *Serratia marcescens* MTCC-97 (*S. marcescens*) were collected from the Microbial Type Culture Collection (IMTECH, Chandigarh, India). The De Man, Rogosa and Sharpe (MRS) agar plate (HiMedia^®^, Mumbai, India) was used for the growth and maintenance of *L. rhamnosus,* whereas, Luria-Bertani agar (LB) (HiMedia^®^, Mumbai, India) was used for the bacterial pathogens. All the bacterial strains were stored at 4 °C for further use.

### 2.2. Biosynthesis of Silver Nanoparticles (AgNPs) Using L. rhamnosus (AgNPs-LR)

The active culture of *L. rhamnosus* was added into a fresh MRS media and incubated at 37 °C for overnight. Following incubation, the grown culture was centrifuged for 10 min. at 10,000 rpm and 4 °C to collect the culture supernatant. Then, culture supernatant (10 mL) was mixed with 0.1 mM silver nitrate solution (90 mL) and incubated at 30 °C for 24 h in dark condition. Observations were made of the colour change of AgNPs after 24 h of synthesis. As part of the characterization of the AgNPs-LR, UV-Vis, FTIR and TEM analysis were performed [31].

### 2.3. Characterization of AgNPs-LR

#### 2.3.1. Ultraviolet-Vis Analysis

In order to characterize AgNPs-LR, a spectrophotometric analysis was performed as a first step. With a resolution of 1 nm, AgNPs-LR were scanned in the range of 300 to 700 nm [31]. The UV-Vis analysis was further used to determine the size of AgNPs-LR using Haiss equation, d = ln((λSPR − λ0)/L1)/L2. Where λSPR is the wavelength at which maximum absorption occurs, λ0 is the wavelength at which minimum absorption occurs at the start of SPR, L1 and L2 are the values taken from the data fit of TEM vs. UV-Vis, whose values are L1 = 6.53 and L2 = 0.0216 [32].

#### 2.3.2. FTIR Analysis

The potential interaction between the culture supernatant of *L. rhamnosus* and AgNO_3_ was examined using Fourier Transform Infrared spectroscopy (FT-IR) (Bruker^®^, Billerica, MA, USA). The spectra were recorded from 500 to 4000 cm^−1^ [33].

#### 2.3.3. Transmission Electron Microscopy (TEM)

Additionally, TEM measurements were performed on the AgNPs-LR for determining the size and shape. For TEM analysis, a JEM-1400 Plus, Jeol, India was used. By applying the AgNPs-LR sample on a grid made of carbon-coated copper and water content was then evaporated within a vacuum dryer for 1 h, TEM analysis was performed [34].

### 2.4. Antibacterial Activity of AgNPs-LA

The antibacterial activity of AgNPs-LR was tested using the agar well diffusion method against *C. violaceum*, *S. marcescens*, and *P. aeruginosa* [35]. A sterilized swab was used to streak the inoculum of the bacterial culture onto a MHB agar plate. With the help of a sterile Cork Borer, wells were punched and each well was filled with AgNPs-LR. Afterwards, zone of inhibition was determined after 24 h of incubation at 37 °C.

### 2.5. Determination of Minimum Inhibitory Concentration (MIC)

In order to evaluate the antibacterial efficacy of AgNPs-LR, the standard broth dilution assay was performed [36]. A series of two-fold dilutions of AgNPs-LR from 1698.7 µg/mL to 0.10 µg/mL concentrations were used to determine MICs in LB broth with active bacterial culture (10^8^ CFU/mL, 0.5 McFarland standard). Only inoculated broth was used for the control, which was incubated at 37 °C for 24 h. MIC is defined as the lowest concentration of AgNPs-LR at which no visible growth can be seen in the tubes at the end of the experiment. In order to confirm the MIC value, a visual examination of the tubes was performed before and after incubation to determine the turbidity.

### 2.6. Assessment of the Quantity of Violacein Pigment in C. violaceum

Violacein production was quantitatively assessed according to standard procedure [37]. In brief, *C. violaceum* was grown for 18 h without and with varying sub-MIC concentrations of AgNPs-LR at 30 °C. For the separation of the insoluble pigment (violacein) from the bacterial cells, centrifugation of 1 mL of culture was carried out at 10,000 rpm for 5 min. To dissolve the pigment, the cell pellet was resuspended in 1 mL of DMSO and vortexed vigorously for 5 min. To spin down the bacteria debris, the suspension was again centrifuged. The UV-spectrophotometer (UV-2600, Shimadzu, Japan) at 585 nm was used for measuring the absorbance of the supernatant.

### 2.7. Assessment of the Quantity of Prodigiosin Pigment in S. marcescens

The production of prodigiosin pigment was assessed using LB medium according to the standard method [38]. The active culture of *S. marcescens* was added into sterile LB medium with and without AgNPs-LR and grown at 30 °C for overnight. After incubation, centrifugation of 2 mL of grown culture was performed for 10 min. at 10,000 rpm to collect the cell pellet. Acidified ethanol was used to dissolve the obtained cell pellet (96 mL ethanol + 4 mL 1 M HCl) by vigorous shaking at room temperature. After centrifuging the sample once again to remove debris, the absorbance of the supernatant was measured at 534 nm using a spectrophotometer (UV-2600, Shimadzu, Japan).

### 2.8. Assessment of QS-Mediated Virulence Factors of P. aeruginosa

#### 2.8.1. Estimation of Pyocyanin

The synthesis of pyocyanin by *P. aeruginosa* was evaluated in LB broth with (sub-MICs) and without AgNPs-LR [39]. The active culture of *P. aeruginosa* was inoculated into a sterile LB medium with and without AgNPs-LR and incubated for overnight at 30 °C. To collect the culture supernatant, 5 mL of grown culture was centrifuged for 10 min. at 10,000 rpm after incubation. Chloroform (3 mL) was then used to extract the pyocyanin from the culture supernatant. The organic phase was collected and further extracted with 1.2 mL of 0.2 N HCl. At last, the absorbance of aqueous phase was taken at 520 nm via spectrophotometer (UV-2600, Shimadzu, Japan).

#### 2.8.2. Assessment of Pyoverdine

As per the standard procedure, the levels of pyoverdine were analysed via performing the standard method [40]. The *P. aeruginosa* was grown overnight at 37 °C without and with sub-MIC amounts of AgNPs-LR. A centrifugation process was performed to obtain a supernatant that was free of cells. Then, 900 µL of 50 mM Tris-HCl (pH 7.4) was mixed with 100 µL of culture supernatant. A multi-mode microplate reader was used to measure the fluorescence emission signal (460 nm) of the sample.

#### 2.8.3. Assessment of LasA Staphylolytic Assay

The *P. aeruginosa* culture supernatant was applied to boiled *S. aureus* cells to determine LasA protease activity [41]. To collect the cell pellets from *S. aureus*, centrifugation of culture was carried out at 8000 rpm for 5 min. After collecting the cell pellet, 0.02 M Tris-HCl (pH-8.5) was added and boiled for 10 min. After that, to adjust the absorbance of 0.8 at 595 nm, it was diluted with 0.02 M Tris-HCl. In the following step, a diluted suspension of *S. aureus* was added to the culture supernatants of *P. aeruginosa* grown with and without AgNPs-LR (sub-MICs) (9:1). Afterwards, the absorbance was measured at 595 nm.

#### 2.8.4. Assessment of LasB Elastinolytic Activity

The measurements of elastolytic activity were carried out using the procedure mentioned by Adonizio et al., (2008) [42]. The method was based on the treatment of a grown *P. aeruginosa* culture with or without AgNPs-LR (sub-MICs). The supernatant was collected and added to 900 µL of a buffer containing 20 mg of elastin congo red (100 mM Tris, 1 mM CaCl_2_, pH-7.5), containing 20 mg of elastin (Sigma^®^, Bengaluru, India). To remove the insoluble components (elastin Congo red), centrifugation was performed after 3 h of incubation at 37 °C. The absorbance of supernatant was measured at 495 nm.

#### 2.8.5. Assessment of Rhamnolipid

The active culture of *P. aeruginosa* was added into sterile LB broth in the presence or absence of AgNPs-LR and incubated at 37 °C for 24 h. Ethyl acetate evaporation was used to extract rhamnolipids from culture supernatant. Using the modified method of Pinzon and Ju (2009) [43], an estimate of *P. aeruginosa* rhamnolipid production was made by dissolving the extracted rhamnolipid in chloroform. A suspension of rhamnolipids was prepared by adding 200 µL of methylene blue solution (0.025%) to 2 mL of solution. A vortexing step was performed for 5 min. and the mixture was incubated at room temperature for 15 min. For phase separation, 0.2 N HCl was added to new tubes containing the chloroform layer after incubation. The mixture was mixed well and left at room temperature for 10 min. By using 0.2 N HCl as blank, 200 µL of the acidic phase holding methylene blue was spectrophotometrically measured at 638 nm.

### 2.9. Determination of the Swimming Motility of P. aeruginosa and S. marcescens

The LB plate containing sub-MIC (1/2 MIC) of AgNPs-LR were spotted with 5 µL of the active culture of *P. aeruginosa* and *S. marcescens*. The control plate was not amended. Incubation was carried out overnight at 30 °C. At the end of incubation, swimming zone was observed in the control and treatment plates [44].

### 2.10. Assessment of Antibiofilm Activity

The glass test tubes were used to determine the antibiofilm effect of AgNPs-LR as the hydrophilic surface [45]. Briefly, sterilized LB medium (3 mL) was transferred to tubes containing 1 mL of active bacterial culture and 500 µL of AgNPs-LR (sub-MICs). In the following step, the contents of the tube were mixed thoroughly and then tubes were incubated at room temperature in a shaker for 72 h. After incubation, the planktonic cells were removed from the tubes and washed with PBS. Then, the formed biofilm inside the tubes was stained with crystal violet. After removing the excess dye via washing with PBS, the stained biofilm was dissolved in acetic acid and absorbance was determined using a spectrophotometer at 595 nm. As a control for the growth of biofilms, LB medium containing individual bacterial strains was used. Estimates of the percentage inhibition of biofilm was made as follows:

O.D. _control_ − O.D. _test_/O.D. _control_ × 100

### 2.11. Light Microscopic Assessment of Biofilm Inhibition

In brief, 60 µL of active culture of bacteria was added in 6-well plates consisting 3 mL of sterile culture medium and allow to grown overnight. Sterile glass coverslips (1 × 1 cm) were placed in the wells along with the respective sub-MIC values of AgNPs-LR. No treatment was administered to the control group. Following incubation of 24 h, the planktonic cells were removed via washing with PBS and then air dried at room temperature for 20 min. The biofilm was stained with crystal violet for 15 min. Afterwards, the slides were washed and air dried for 30 min. to remove any excess dye. In order to visualize the biofilms, a light microscope was used (Axioscope A1, Zeiss, Jena, Germany). Magnification was set at 40× for the images [46].

### 2.12. Assessment of Exopolysaccharide (EPS) Production

A staining assay using ruthenium red was conducted to determine AgNPs-LR activity in reducing EPS content in biofilms [47]. Each of the bacterial test strains (100 µL) and AgNPs-LR were incubated for 24 h at 37 °C. After incubation, planktonic cells were removed and the cell pellet was washed with sterile PBS (200 µL). In order to stain the biofilms formed by the adherent cells, 200 µL of ruthenium red (0.01%) (Sigma-Aldrich^®^, Bangalore, India). A well that was free of biofilm and a well containing ruthenium red served as a blank. For the next step, the plate was incubated for 1 h at 37 °C. The residual stain was then re-dispersed into a new microtiter plate and the absorbance measured at 450 nm. Amount of dye fixed to biofilm matrix measured as follows:

Abs_BF_ = Abs_B_ − Abs_S_

where, Abs_B_ = absorbance of the blanks, and Abs_S_ = absorbance of the residual stain collected from the sample wells.

## 3. Results

### 3.1. Synthesis and Characterization of AgNPs-LR

**Figure 1:**
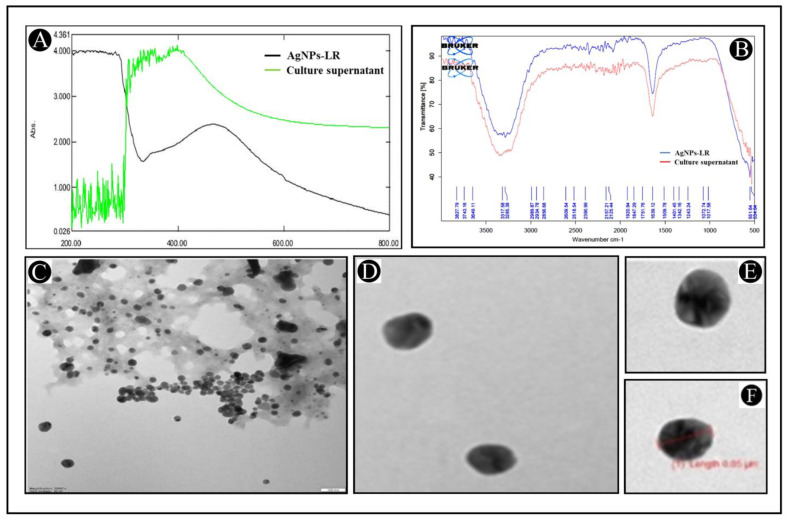
Characterization of AgNPs-LR. (**A**) UV-visible absorption spectra of AgNPs-LR. (**B**) FT-IR analysis of AgNPs-LR. (**C**–**F**) Morphological analysis of AgNPs-LR via TEM analysis.

As a result of adding 1 mL of culture supernatant into 10 mL of 0.1 mM silver nitrate solution (1:10), the biosynthesis of AgNPs-LR has been achieved. It was observed during the period of incubation that the colour changed from yellow to dark brown, while the intensity increased over the course of the period of incubation, as a result of a successful biosynthesis of AgNPs-LR. As a first step, UV-Vis analysis of AgNPs-LR was conducted in order to confirm their biosynthesis. According to spectroscopic measurements made after 24 h of synthesis of AgNPs-LR, absorption spectrum with a clear symmetry was observed with a highest absorption at 464 nm (Figure 1A). Bu using information available from the UV-Vis analysis and Haiss equation, we calculated the size of AgNPs-LR as 6.26 nm. By using FTIR spectrum, different functional groups can be identified, as a result of which silver ions are reduced and stabilizing silver nanoparticles. A spectrum of AgNPs-LR is shown in Figure 1B. As shown in the figure, there are several vibrational bands that can be observed in the spectrum, which indicates the existence of several functional groups. The vibrational bands at 3285.38 cm^−1^, 2934.78 cm^−1^, 2125.44 cm^−1^, 1639.12 cm^−1^, 1401.45 cm^−1^, 1243.24 cm^−1^ and 551.64 cm^−1^ are hydroxyl, C-H/methylene, thiocyanate, alkanyl, carboxylate, aromatic ethers, respectively. Study of infrared spectroscopy suggested the predicted factor groups bind to metals with the most strength, and by coating particles, they can be prevented from agglomerating and maintained for a prolonged period of time. Furthermore, the TEM studies of the AgNPs-LR revealed that most of the nanoparticles were spherical and polyhedral in shape, as well as poly dispersed nanoparticles (Figure 1C–E). It was found that the diameter of the AgNPs-LR ranged from 5 to 70 nm.

### 3.2. Antibacterial Potential of Biosynthesized AgNPs-LR

**Figure 2:**
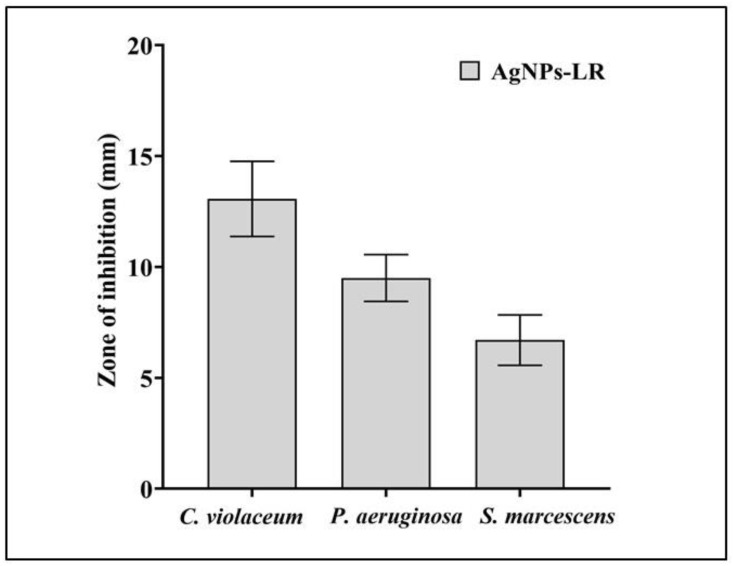
Antibacterial activity of AgNPs-LR against different Gram-negative bacterial pathogens. Values are represented as the mean ± SD of three independent experiments.

Biosynthesized AgNPs-LR shown to possess antibacterial properties against all of the tested bacterial strains in well diffusion assays. Among the tested bacteria, *C. violaceum* showed the highest zone of inhibition, followed by *P. aeruginosa* and *S. marcescens* Figure 2. Additionally, a broth microdilution was employed in order to determine the MIC of AgNPs-LR. The AgNPs-LR had MIC values of 13.27 μg/mL against *C. violaceum*, 26.54 μg/mL against *P. aeruginosa* and 53.08 μg/mL against *S. marcescens*, respectively. In order to test the efficacy of biosynthesized AgNPs against formation of biofilm as well as QS-regulated virulence factors, the concentrations of AgNPs were below the inhibitory concentrations (sub-MICs).

### 3.3. Inhibition of Inhibition of Virulence Factors of C. violaceum

**Figure 3:**
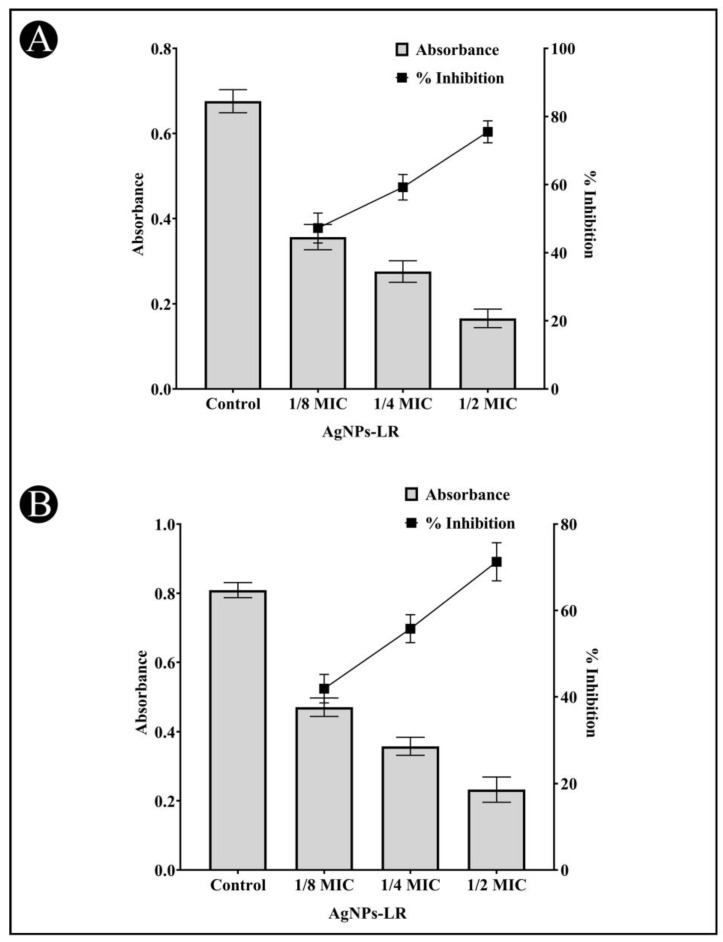
Anti-QS activity of AgNPs-LR against *C. violaceum* and *S. marcescens*. (**A**) An analysis of the quantitative inhibition of violacein in *C. violaceum* using AgNPs-LR. (**B**) An analysis of the quantitative inhibition of prodigiosin in *S. marcescens* using AgNPs-LR. Values are represented as the mean ± SD of three independent experiments. A secondary *y*-axis shows the percentage inhibition.

The AgNPs-LR has been checked for their preliminary anti-QS activity by determining their impact on *C. violaceum* pigment production. The pigment production in this strain is controlled by QS. Reduced pigment production can be considered as an indication of the presence of anti-QS activity. A treatment with 1/2, 1/4 and 1/8 MIC of AgNPs-LR in *C. violaceum* resulted in a 75.49%, 59.22% and 47.23% reduction in the synthesis of violacein, respectively (Figure 3A). This clearly indicates that green synthesized AgNPs-LR are capable of exhibiting anti-QS activity.

### 3.4. Inhibition of Virulence Factors of S. marcescens

AgNPs-LR were also tested for anti-QS activity against *S. marcescens* in an effort to determine whether they inhibit the broad spectrum of QS. *S. marcescens* produces a pink-red pigment called prodigiosin that is regulated via QS. As per Figure 3B, a range of sub-MICs of AgNPs-LR were found to reduced production of prodigiosin in *S. marcescens*. At the concentration of 1/2, 1/4 and 1/8 MIC, AgNPs-LR led to a 71.28%, 55.78% and 41.90% inhibition of prodigiosin, respectively.

### 3.5. Inhibition of Virulence Factors of P. aeruginosa

**Figure 4:**
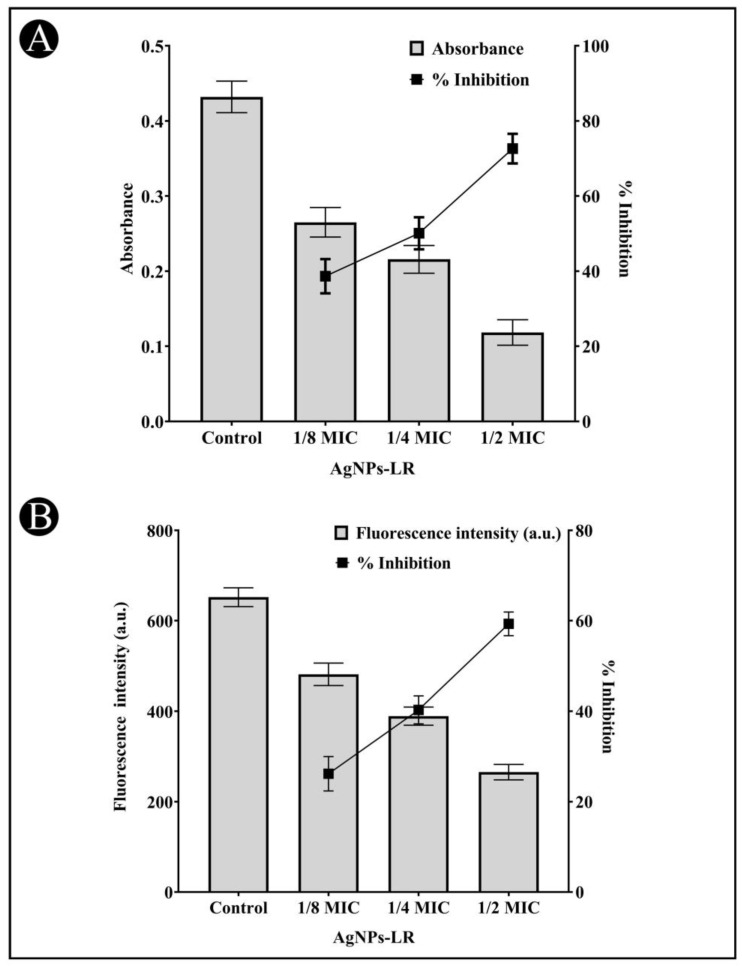
Anti-QS activity of AgNPs-LR against *P. aeruginosa*. (**A**) An analysis of the quantitative inhibition of pyocyanin production in *P. aeruginosa* using AgNPs-LR. (**B**) An analysis of the quantitative inhibition of pyoverdine production in *P. aeruginosa* using AgNPs-LR. Values are represented as the mean ± SD of three independent experiments. A secondary *y*-axis shows the percentage inhibition.

The virulence factor of *P. aeruginosa* mediated by QS was examined against AgNPs-LR. A blue-green pigment pyocyanin is produced by *P. aeruginosa* and is controlled by the communication between bacterial cells. The pigment production of the cells was gradually decreased following treatment with AgNPs-LR. A concentration of 1/2, 1/4 and 1/8 MIC reduced the pigment production of *P. aeruginosa* by 72.60%, 50.07% and 38.65% (Figure 4A). The pyocyanin contained in *P. aeruginosa* has been shown to be a significant contributor to its pathogenic potential through the interference with cellular functions of the host.

A pigment known as pyoverdine can also be produced by several strains of *P. aeruginosa* which are virulent. There was an inhibition of pyoverdine production in the supernatant by 59.30%, 40.28%, and 26.17%, respectively, when sub-MICs of AgNPs-LR is present (Figure 4B).

**Figure 5:**
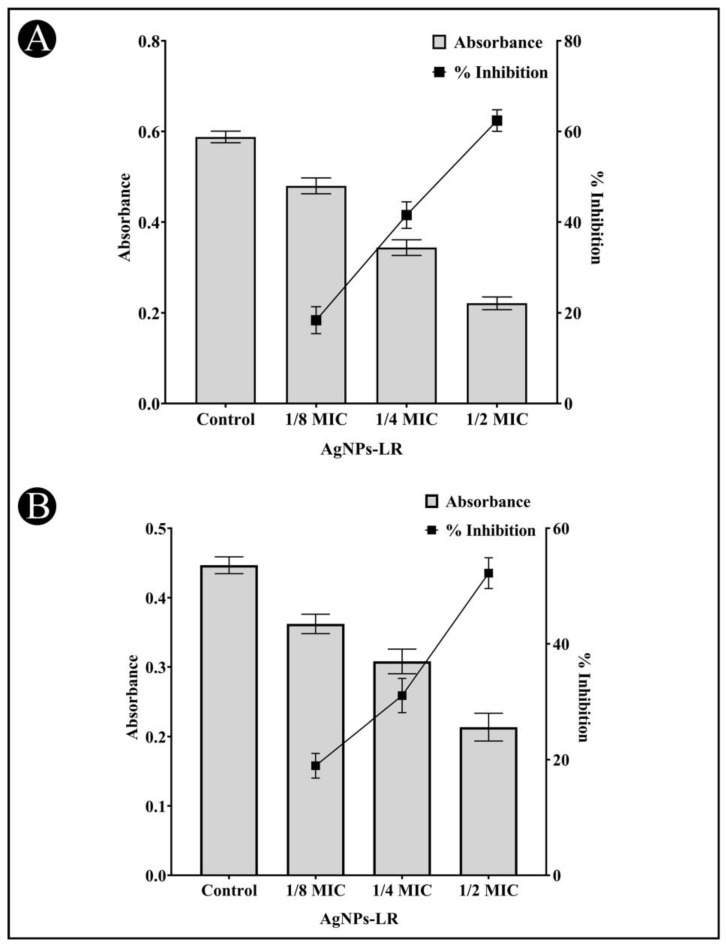
Anti-QS activity of AgNPs-LR against *P. aeruginosa*. (**A**) An analysis of the quantitative inhibition of LasA protease production in *P. aeruginosa* using AgNPs-LR. (**B**) An analysis of the quantitative inhibition of LasB elastase production in *P. aeruginosa* using AgNPs-LR. Values are represented as the mean ± SD of three independent experiments. A secondary *y*-axis shows the percentage inhibition.

A virulent strain of bacteria produces proteolytic enzymes that cause damage to the tissues of the host upon successful infection. A staphylolytic assay was used to determine whether AgNPs-LR inhibit LasA protease activity. After treatment with sub-MICs of AgNPs-LR, LasA protease activity decreased by 62.41%, 41.55%, and 18.36%, respectively (Figure 5A).

An eleastase is a hydrolytic enzyme produced by bacteria during an infection that destroys and inhibit the host immune system. In the presence of AgNPs-LR, *P. aeruginosa* showed a concentration-dependent inhibition of its elastinolytic activity (Figure 5B).

**Figure 6:**
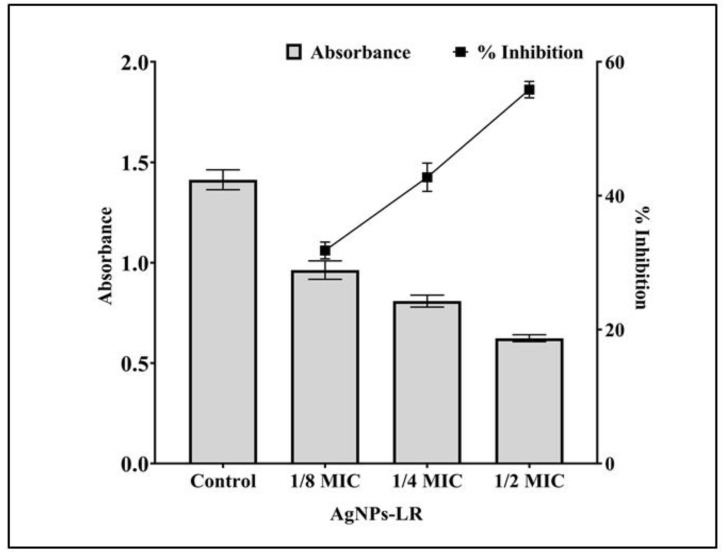
An analysis of the quantitative inhibition of rhamnolipid production in *P. aeruginosa* using AgNPs-LR. Values are represented as the mean ± SD of three independent experiments.

In addition to maintaining the structure of biofilms, rhamnolipids play an important role in adhering bacterial cells to solid surfaces. Rhamnolipid production by *P. aeruginosa* is regulated by RhlR-RhlI QS. Following treatment with AgNPs-LR, rhamnolipid production was reduced (Figure 6). Rhamnolipid production was decreased by 55.86%, 42.76% and 31.82%, respectively, in the presence of sub-MICs of AgNPs-LR.

### 3.6. Quantitative Inhibition of the Formation of Biofilms

**Figure 7:**
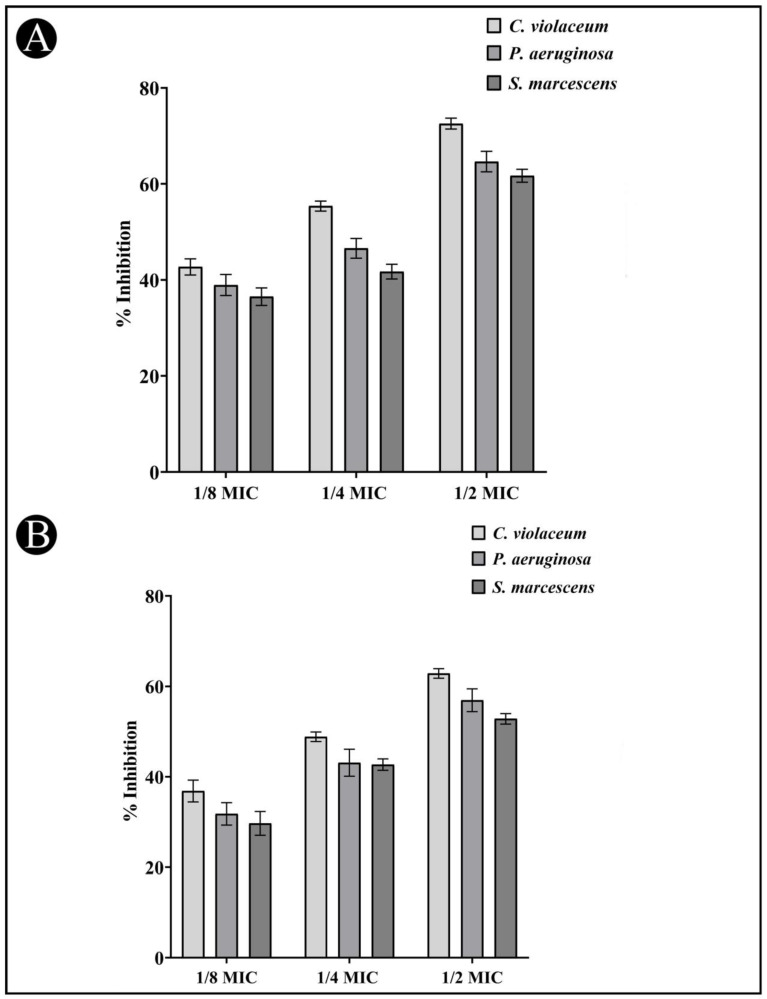
Anti-biofilm and EPS inhibition activity of AgNPs-LR against different Gram-negative bacterial pathogens. (**A**) An analysis of the quantitative inhibition of biofilm production using AgNPs-LR. (**B**) An analysis of the quantitative inhibition of EPS production using AgNPs-LR. Values are represented as the mean ± SD of three independent experiments.

An AI-mediated QS phenomenon is often responsible for regulating the formation of a biofilm by regulating its mechanisms. The results obtained from the study on the effects of the AgNPs-LR on the formation of biofilms is shown in Figure 7A for all three bacteria tested. The development of bio-films of *C. violaceum* was inhibited by 72.56%, 55.37% and 42.70%, when 1/2, 1/4, and 1/8 MIC concentrations were used as treatment. The biofilm of *P. aeruginosa* was inhibited by 64.66%, 46.58% and 38.93% at sub-MICs. Similarly, when sub-MICs are present, the biofilms of *S. marcescens* decreased by 61.70%, 41.72%, and 36.50%, respectively.

### 3.7. Inhibition of EPS Production

The EPS matrix provides protection and support for the biofilm and is critical for its formation, stability, and function. EPS can help bacteria adhere to surfaces, create channels for nutrient flow, and provide protection against antibiotics and other stressors. The present study found that EPS production decreased upon the treatment of AgNPs-LR as 62.84%, 48.84% and 36.83% in *C. violaceum*, 56.91%, 43.12% and 31.77% in *P. aeruginosa* and 52.80%, 42.68% and 29.67% in *S. marcescens* at sub-MICs, respectively (Figure 7B).

### 3.8. Analysis of Biofilm Inhibition on Glass Surfaces Using Microscope

**Figure 8:**
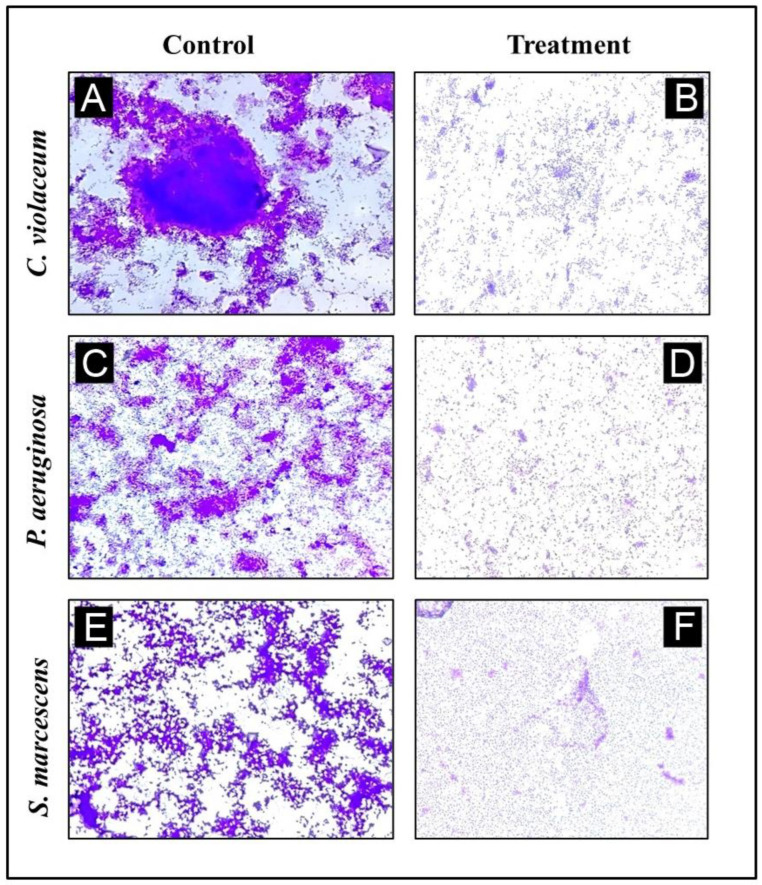
A representative light micrograph of a biofilm showing the effects of AgNPs-LR at their highest sub-MICs. (**A**) Control of *C. violaceum*, (**B**) Treatment of *C. violaceum* with ½ MIC, (**C**) Control of *P. aeruginosa*, (**D**) Treatment of *P. aeruginosa* with ½ MIC, (**E**) Control of *S. marcescens*, (**F**) Treatment of *S. marcescens* with ½ MIC.

Using a microscopy technique, it was possible to perform a further evaluation of the inhibition of biofilms. For the purpose of examining a change in the biofilm architecture, the test bacteria were cultured without and with the maximum sub-MIC of AgNPs-LR in order to visualize biofilm architecture changes. All the tested bacteria displayed a dense cluster of cells on the glass coverslips, as can be seen from the light microscopy images (Figure 8A–E). With AgNPs-LR treatment, cells were seen in a scattered form on the glass surface and were significantly reduced in clustering.

### 3.9. Inhibition of Swimming Motility

**Figure 9:**
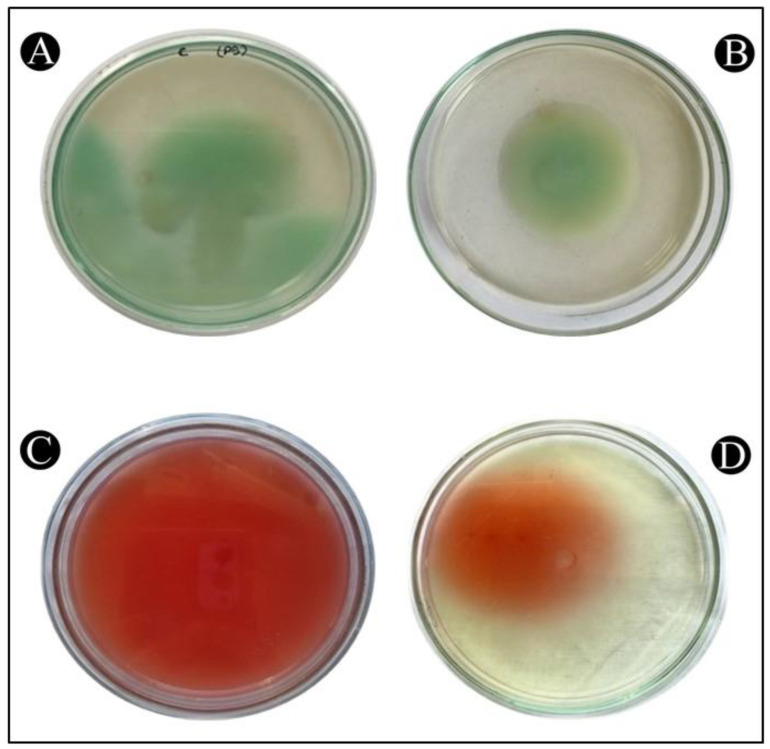
The inhibition of the swimming motility of *P. aeruginosa* and *S. marcescens* by AgNPs-MK. (**A**) Control of *P. aeruginosa*, (**B**) Treatment of *P. aeruginosa* with ½ MIC, (**C**) Control of *S. marcescens*, (**D**) Treatment of *S. marcescens* with ½ MIC.

QS has a very important role in controlling the movement of *P. aeruginosa* and *S. marcescens*, a crucial factor in determining the spread of infection in a host. It is also considered as a crucial factor in the pathogenicity of both bacteria, so it is an important factor in virulence. In Figure 9A–D, it is shown that the control *P. aeruginosa* and *S. marcescens* swims across the entire Petriplate after overnight incubation. Whereas, there was a decrease in the zone of swimming in the treatment plates.

## 4. Discussion

In order to synthesize metal nanoparticles, there are a variety of approaches such as chemical, physical and biological methods that can be utilized. There are significant drawbacks associated with chemical synthesis, primarily the fact that dangerous and non-biodegradable by-products are formed, making them harmful for the environment. The concept of green synthesis is gaining increasing popularity because of its environmental benefits, such as minimizing waste production, using non-hazardous materials and enhancing its environmental friendliness [19]. Besides not causing disease, probiotic bacteria also prevent pathogenic bacteria from multiplying in animals’ digestive system and increase intestinal microflora that is beneficial for the animal. There has been a growing market for probiotics in the majority of countries [48].

Using *L. rhamnosus* as a basis for the production of AgNPs, the purpose of the present study was to establish a simple, green, and inexpensive approach that could be utilized to synthesize AgNPs. A prepared sample of *L. rhamnosus* was transferred to a final concentration of 1 mM of silver nitrate in order to observe the formation of AgNPs-LR. The colour change in the sample indicated AgNPs-LR formation, which were then characterized using UV-Vis, FT-IR and TEM analysis. Analyzing AgNPs with UV-Vis is a common technique used to characterize the optical properties of these nanoparticles. The technique is based on the principle of interaction of light with the localized surface plasmon resonance of AgNPs [49]. Therefore, the electron transition that occurs at 464 nm during AgNPs synthesis can be interpreted as a result of the interaction between incident light and the localized surface plasmon resonance of the AgNPs. At 464 nm, the absorption peak suggests that the electron transition involved is primarily a dipole transition. In a dipole transition, the conduction electrons of the AgNPs are excited from the ground state to a higher energy state by absorbing a photon with a specific energy corresponding to the wavelength of 464 nm [50,51,52]. FT-IR analysis of AgNPs is a technique utilized to determine the chemical composition and bonding properties of these nanoparticles. This technique is based on principle of the interaction of infrared radiation with the molecular vibrations of the nanoparticles [53]. Whereas, TEM analysis was performed to characterize the size, shape and distribution of synthesized AgNPs.

Previously, an iron oxide nanoparticle has been synthesized by Torabian et al. [54] using the green synthesis approach. Upon synthesis, iron oxide nanoparticles were found to be around 15 nm in size. Nanoparticles had round to spherical shape and they found its application in drug delivery or therapy as safe, effective and inexpensive [54]. By synthesizing AgNPs from the supernatant filtrate of *L. acidophilus*, Rajesh et al. [55] designed eco-friendly antibacterial components. Using electron microscopy, they determined that the particles had spherical shapes with sizes ranging from 4–50 nm. According to their report, AgNPs showed antibacterial properties when used against *Klebsiella pneumoniae* by causing cytolysis and destroying the membrane of the bacterial cell [55]. A further study conducted by Nithya et al. [56] examined the antimicrobial efficacy of AgNPs synthesized from *Brevibacterium linens* against multidrug-resistant clinical isolates and demonstrated that these nanoparticles were highly effective. As a result of the AgNPs incubated with *Escherichia coli* colonies for 3 h, it was observed that viable cells had decreased. In contrast, it can cause *Staphylococcus aureus* inhibition zones similar to those of Amikacin. Selenium nanoparticles (SeNP) with 50–80 nm size was prepared by Xu et al. [57] using *L. casei*. Furthermore, in mice infected with Enterotoxigenic *E. coli* K88, SeNP treatment resulted in lowering inflammatory cytokines and oxidative stress in the mice [57]. Using the probiotic *L. kimchicus* DCY51 isolated from traditional Korean kimchi, Markus et al. [58] synthesized gold nanoparticles (AuNPs). Synthesized AuNPs were found to be spherical structure with a size between 5–30 nm. The antioxidant properties of AuNP were demonstrated by its ability to remove free radicals [58]. The antimicrobial efficacy of AgNPs synthesized from one more probiotic *L. amylophilus* GV6 has been tested by Kumar et al. [59] using agar well plate assay against several bacterial pathogens such as, *B. subtilis* MTCC 121, *S. aureus MTCC 96*, *P. aeruginosa* MTCC 424, *K. pneumoniae* MTCC 109 and *E. coli* MTCC 43, which reported the 1.5 cm inhibition zone against *S. aureus* MTCC 96. Using two probiotic bacteria, *L. acidophilus* 58p and *L. plantarum* 92T, Garmasheva et al. [60] synthesized AgNPs and studied their antibacterial activity. The AgNPs obtained from *L. acidophilus* 58p were found more active against *E. coli*, *S. epidermidis*, *S. flexneri*, *K. pneumonia*, and *S. sonnei* than *L. plantarum* 92T AgNPs. In a similar study, Naseer et al. [61] synthesized AgNPs from *L. bulgaricus* and evaluated their antibacterial effectiveness against *S. aureus*, *S. epidermidis* and *S. typhi*. Gram-negative bacteria were found to be more susceptible than Gram-positive bacteria. A recent study by Sharma et al. [62] also synthesized safe, inexpensive and biocompatible AgNPs using four different probiotic isolates such as, *L. plantarum* F22, *L. paraplantarum* KM1, *L. pentosus* S6 and *L. crustorum* F11. Various bacterial and fungi pathogens viz., *B. cereus, L. monocytogenes*, antibiotic-resistant *S. aureus*, *P. aphanidermatum*, *P. parasitica* and *F. oxysporum* were also found to be susceptible to these AgNPs. Among them, AgNP synthesized by *L. crustorum* F11 showed maximum inhibition against all pathogens, with maximum activity against *S. aureus* (20 ± 0.61 mm) and *F. oxysporum* (23 ± 0.37).

The current study further explores the antibacterial, antibiofilm and anti-QS activities of biosynthesized AgNPs-LR. As a first step, biosynthesized AgNPs were tested against different bacteria pathogens using well diffusion assays. According to our results, the biosynthesized AgNPs-LR display the highest antibacterial activity against *C. violaceum*, *S. marcescens* and *P. aeruginosa*, respectively. As reported earlier, the high antibacterial activity of biosynthesized AgNPs resulting from reactive oxygen species production and the damage to membranes [63,64]. By virtue of the low MIC of biosynthesized AgNPs against bacterial pathogens, it is not necessary to use conventional antibiotics in conjunction with the biosynthesized AgNPs. Additionally, biosynthesized AgNPs showed higher susceptibility to *C. violaceum* and *P. aeruginosa* than *S. marcescens*. In general, each of these bacteria is capable of forming biofilms, which are difficult to eradicate even with conventional antibiotics available on the market, since their extracellular matrix also reveals resistance to both environmental factors and antibiotics [65,66]. The formation of biofilms contributes to pathogenesis, with almost 80% of infections attributed to biofilm formation [17]. Biofilms are responsible for reducing the efficiency of antibiotics by up to a thousand-fold, which places a burden on the health care system to treat infections. The AI often contributes to QS that controls the formation of a biofilm in a given environment. The synthesized AgNPs-LR were tested for their effect on the development of biofilms in all three bacterial pathogens and the results showed that they effectively inhibited the formation of biofilms at sub-MIC levels. It has been discovered that bacteria that form biofilms resist both chemical and physical therapy and their virulence genes are coordinated in their expression [67]. Previous studies found tobramycin resistance in biofilms of *P. aeruginosa* to be 1000 times higher than in planktonic bacteria [68]. A study carried out using AgNPs derived from the bark extract of *Holarrhena pubescens* reported that the AgNPs inhibited the development of biofilms of imipenem-resistant *P. aeruginosa* [69]. Additionally, AgNPs also inhibited the biofilm development of methicillin-resistant *S. aureus* and *P. aeruginosa* producing extended-spectrum beta-lactamases (EsbL) [70]. Histidine-capped AgNPs were also known to eradicate mature biofilm of *K. pneumoniae* by Chibber et al. [71].

The bacterial QS process is a unique way in which bacteria communicate between themselves, through cell density can be sensed by bacteria in their adjacent atmosphere and this in turn results in the activation or suppression of specific genes in the bacteria [72,73]. QS-dependent gene expression is responsible for many important bacterial characteristics such as physiology, virulence, and the formation of biofilms in bacteria. A huge attention has been given to research on QS because of its potential for human medicine over the past 15–20 years [74]. Thus, the current study also examined an inhibitory effect on QS of AgNPs-LR against *C. violaceum*, *P. aeruginosa* and *S. marcescens*.

Violacein production by *C. violaceum* is regulated by the QS system based on the density of bacteria in the population. Although, violacein itself is not usually considered a pathogenic factor, infections caused by *C. violaceum* can be serious and even life-threatening in individuals with compromised immune systems [75]. In these instances, violacein may help bacteria evade the immune system and establish infection by contributing to their virulence. In spite of the fact that cell-to-cell communication is critical to bacterial physiology and virulence, QS inhibitors have been shown to inhibit the production of violacein by *C. violaceum*, thus providing insights into the potential of QS inhibitors as therapeutic agents against bacterial infections [76].

Pyocyanin production in *P. aeruginosa* is also regulated by QS, just as violacein production. Pyocyanin is a blue-green pigment that is crucial for the pathogenesis of *P. aeruginosa* infections [77]. Pyocyanin plays a key role in *P. aeruginosa* infections by causing oxidative stress in the host. The pyocyanin pigment damages host cell membranes and contributes to the destruction of host tissues by generating reactive oxygen species (ROS). Aside from causing tissue damage and exacerbated infection, oxidative stress triggers inflammation [78]. The immune response to infection has also been interfered with by pyocyanin. Immune cells such as neutrophils and macrophages, which fight bacterial infections, are inhibited by it. As a result, *P. aeruginosa* infection that evades the immune system can become persistent [79,80]. Additionally, *P. aeruginosa* produces pyocyanin, a fluorescent green-yellow siderophore [81]. By facilitating iron acquisition, promoting bacterial growth, and stimulating the host immune response, it also plays an immense part in the pathogenesis of *P. aeruginosa* infections [82]. Furthermore, *P. aeruginosa* infections involve the production of a protease enzyme called Las A. Among the key factors in the pathogenesis of *P. aeruginosa* infection, it degrades host tissue proteins, interferes with host cell signaling, stimulates host immune responses, and promotes biofilm formation and detachment [83]. As for Las B, it is a zinc metalloprotease enzyme produced by *P. aeruginosa* that degrades host tissue proteins, including elastin, collagen, and fibronectin, during pathogenesis. This can result in tissue damage and destruction, which is then used by the bacterium to spread and colonize new sites within the host as a result of the damage [84]. Additionally, *P. aeruginosa* produces surface-active molecules known as rhamnolipids. Moreover, *P. aeruginosa* infections involve the activity of these molecules in a variety of ways. During infection by *P. aeruginosa*, it contributes to host cell lysis, promotes biofilm formation, stimulates the immune response, and aids bacterial adaptation to stress [85]. The QS is also responsible for the synthesis of prodigiosin, a bright red pigment produced by *S. marcescens*. The prodigiosin contributes to the pathogenesis of *S. marcescens* infections, as it is majorly responsible for the formation of biofilm, antimicrobial activity, immunomodulation, and cytotoxicity [86]. It is therefore possible to gain insights into the infections caused by pathogenic bacteria by regulating the activity of QS.

Furthermore, movement of bacteria by swimming is known as swimming motility that involves the rotation of flagella to propel the cell through liquid environments. Swimming motility plays a crucial role in the formation and spread of biofilms, as well as in the regulation of QS signaling pathways that are important for bacterial communication and virulence. Inhibition of swimming motility has therefore raised as a possible way for the development of antibiofilm and anti-QS agents. EPS inhibition is also an emerging strategy for development of antibiofilm and anti-QS agents [87]. The EPS matrix plays an important role for the development of biofilms, providing mechanical stability, protection against environmental stresses, and a barrier to antimicrobial agents. By targeting the EPS matrix or QS signaling pathways, it may be possible to prevent biofilm formation, make biofilms more susceptible to antimicrobial agents and disrupt a wide range of bacterial behaviors [88]. Accordingly, the results of this study clearly indicated that AgNPs-LR synthesized from *L. rhamnosus* played a broad-spectrum antibiofilm and anti-QS activity against Gram-negative pathogenic bacteria.

## 5. Conclusions

The biosynthesized AgNPs-LR demonstrated a remarkable reduction in multiple QS-regulated functions in Gram-negative bacteria, such as, *C. violaceum*, *P. aeruginosa*, and *S. marcescens*. In *C. violaceum*, there has been a significant decrease in the production of violacein pigments of more than 70%. Upon treatment with AgNPs-LR, *S. marcescens* virulent trait controlled by QS was also reduced by 70%. A dose-dependent inhibition of *P. aeruginosa* virulence factors was also observed with AgNPs-LR. All test bacteria were found to show a decrease in their ability to form biofilms at their respective sub-MICs by a dose-dependently manner. In addition, there was a notable reduction in the formation of biofilms on the surfaces of the coverslips, swimming motility as well as the production of EPS. As a result, it can be concluded that biosynthesized AgNPs-LR could be exploited for the treatment of skin infections resulting from topical application. In addition to this, medical implants/devices can also be coated with these materials in order to inhibit the bacterial adhesion and to prevent the formation of biofilms on the surfaces. However, there is a need to perform more *in-vivo* studies to determine the therapeutic efficacy of AgNPs-LR against infections caused by pathogens that are resistant to known antibiotics.

## Data Availability

All data generated or analyzed during this study are included in this article.
